# Study of the Seismoelectric Effect of the Second Kind Using Molecular Sensors

**DOI:** 10.3390/s21072301

**Published:** 2021-03-25

**Authors:** Vadim Potylitsyn, Danil Kudinov, Alekseev Dmitry, Ekaterina Kokhonkova, Sergey Kurkov, Ivan Egorov, Aleksandra Pliss

**Affiliations:** 1 Laboratory of Electroacoustics, Siberian Federal University, 660041 Krasnoyarsk, Russia; kudinovdanil@yandex.ru (D.K.); kokhonkova@yandex.ru (E.K.); 2 Laboratory of the Geophysical Research of the Arctic and Continental Margins of the World Ocean, Phystech School of Radio Engineering and Computer Technology, 141700 Dolgoprudny, Russia; alexeevgeo@gmail.com (A.D.); kurcov@mail.ru (S.K.); egorov.ivan83@gmail.ru (I.E.); pliss.alex@mail.ru (A.P.); 3 Laboratory of the Tectonic-Electromagnetic Interactions, Schmidt Institute of Physics of the Earth, 123995 Moscow, Russia

**Keywords:** seismoelectric, sensors, gas condensate field, molecular-electronic geophones

## Abstract

The article is devoted to the study of the potential possibilities of using molecular-electronic sensors of seismic waves for field work using the seismoelectric method to explore the hydrocarbon deposits. The introduction provides an analytical review of the current state of research based on data from science magazines and patents. It is shown that at present, seismoelectric effects are at the stage of experimental implementation into the practice of field work for oil and gas geophysical prospecting. Further in the article, theoretical estimates and results of mathematical modeling of the manifestation of seismoelectric (SE) phenomena in the regions of hydrocarbon anomalies are presented, numerical estimates of the values of the seismic and secondary electromagnetic fields are given. The analysis of the results (on a tank and real gas condensate field) showed that the use of molecular-electronic geophones, which have a higher sensitivity and operate in a wider frequency range (up to 0.1 Hz), allows one to obtain higher signal-to-noise ratio. Thus, it has been experimentally established that the use of molecular sensors for recording seismic electric effects when searching for deposits is more preferable when carrying out field work.

## 1. Introduction

The interaction of seismoacoustic and electromagnetic fields at the subsurface structural interfaces or in ion-conducting media was described back in the 1940s. The first to pay attention to these phenomena in rocks was the Soviet scientist Ivanov A. G. Later, the theoretical model was developed by Soviet and American researchers in 1944, Frenkel J. I. [1] and Biot M. A. in 1956 [2], respectively, who described the effect of the electromagnetic (EM) field generation when the crystalline rock was exposed to elastic mechanical vibrations.

To date, a number of research papers on the SE method have been published, covering its theoretical and practical aspects [3]. More recent theoretical studies have included the pioneering approach of solving the fully coupled mechanical-electrodynamical problem [4], and recognition of the importance of slow Biot waves in SE conversion [5]. The modern state of the method, with many details on theory, modeling, and applications, has been described in comprehensive reviews [6,7,8]. Less common SE applications include planetary-scale analysis of electrokinetic fields caused by lunar tides [9]. It should be noted that most of the analysis has been done in the frequency domain.

The seismoelectric effect (SSE) is considered in more detail in [8,10,11,12]. The authors mainly considered the seismic-electric effect induced by a seismic wave using a non-explosive source of the seismic field. If we consider theoretical modeling, then it concerns sufficiently high frequencies from 10 to 100 kHz, for example, in their work, Ref. [8] gave theoretical modeling and calculations as well as experimental data on a tank with sand and an electrolyte. Others [13,14] cite simulation data for frequencies above 20 kHz and indicate that with an increase in the frequency of the seismic field, the amplitude of the induced electromagnetic field will become smaller. For the frequency range 0.1–100 Hz, there is no adequate developed mathematical model. The closest works [15,16] considered the frequency of 400 Hz. This range is interesting because many of the expert data obtained by different authors on real hydrocarbon deposits were obtained for the frequency range 0–100 Hz [17] and the authors [18] indicated that in this frequency range, it manifests itself more strongly, which may be associated with the frequency characteristics of the geological section and the predominance of low-frequency components in the received seismic signal. In the work [19], the author summarizes modern concepts of seismoelectric effects in coloid solutes and electrolytes, and conducts experimental studies in the ultrasonic range in laboratory conditions on coloid solutes. In this work, the author gives formulas for calculating the electrical potential of electrolytes in the low-frequency range under impulse exposure. Similar results were obtained by [20]. The seismoelectric method is considered mainly using a seismic field generator and the calculated ratios for the poroelastic medium have already been obtained; for the passive version of the seismoelectric method, when no artificial sources of the field of the derived ratios are used, the authors of this article could not find references to the use of such a measurement technique in scientific publications.

Field observations of seismoelectric effects are given in [17], where a source with a 35 ton impact force was used to generate seismic vibrations, and measurements were made using a seismic streamer. The authors described that the convergence of experimental and calculated data was observed. According to the authors, seismoelectric effects were observed mainly at frequencies up to 100 Hz.

Furthermore, Berg A. et al. [21], and Melnikov, Bobrovnikov et al. [22] carried out a fairly large amount of research on the seismoelectric (SE) method of the geophysical exploration of hydrocarbon deposits in the offshore areas of the Gulf of Mexico and the Barents Sea. The results showed that the active SE method allowed for the detection of hydrocarbon anomalies with a probability of at least 70%, which is almost twice as high as the results obtained by existing alternative seismic exploration methods. The use of the active version of the SE method described in [21,22] is associated with large labor costs and the use of bulky and powerful sources of EM and seismic fields.

There are also developments concerning the application of the SE phenomena for geophysical exploration of hydrocarbon deposits by means of the impact on the formation of noise seismoacoustic (SA) fields emitted by drilling rigs during drilling and the interaction of these noises with the natural Earth EM field. In [18], the results of studies are given, which, in the opinion of the authors, will make it possible to abandon the sources of both SA and EM fields only by observing passive seismoelectric effects. Only the frequency range up to 100 Hz has been analyzed. The use of this technology is possible both at the drilling stage and at the stage of primary exploration. It is shown that the use of external additional sources of seismic radiation only enhances the SE effect observed on the Earth’s surface. The authors estimated the depth of sounding for this method at 300 m and the registration of the EM field was organized using both magnetic antennas and active sensors of telluric currents.

The patent in [23] describes the specialized sensors for recording the SE effects. The authors point out that the application of this sensor system is possible for a completely passive SE method, that is, for the case when only natural fields are recorded. The schemes of sensors for the registration of the EM field are given. In the patent of [24] by the same authors describing the implementation of the SE method of searching for hydrocarbons, it is indicated that the frequency range is limited up to 100 Hz. However, nothing was said about the lower end of the frequency range.

Patent [25] proposed a hydrocarbon search system using seismic focusing and electrotomography. In their opinion, focusing the SA field in a certain region of space should allow for obtaining additional SE data and interpreting them in more detail. The authors of the patent concluded that the combination of an active system of seismic prospecting and electrical prospecting in the variant with a focus on the seismic field will make it possible to carry out measurements with the lowest time consumption. The use of this system can only be applied in explored fields to clarify the data on the structure of productive strata and their sizes.

The system for the passive SE method is given in the patent in [26]. According to the authors, the information on the SE effects in a productive hydrocarbon reservoir can be contained in the envelope of the SE signal phase as well as in other characteristics. In particular, the signals are noted to possibly be amplitude, frequency, or phase modulated. The authors pointed out that the observed seismic and EM signals could be recorded with a time delay. This system consists of seismic and electromagnetic field sensors (in this case, the sensors can be integrated) and the information processing system using processor technology. It should be noted that the authors did not provide algorithms for calculating or estimating the parameters of the SE effects.

The authors of this article previously conducted relevant experiments and showed that using the SE effect could be used to detect hydrocarbon deposits on land, both in passive application and in semi-active performance [27,28], that is, without using an active source of the EM field. This approach can significantly reduce the weight and size indicators and, as a result, the cost of exploration work.

The reviewed publications describe various schemes for measuring the SE effects, which can be classified as follows:

- Active systems with sources of excitation of EM and seismic vibrations;
- Semi-active systems. In this case, only one type of wave is emitted: EM or seismoacoustic; and
- Completely passive, when only natural EM fields and microseismic vibrations are recorded without the external excitation of EM or seismic waves.

Almost all authors indicate that the recorded frequencies are in the range of up to 100 Hz. This is due to the fact that the EM field, when propagating through a continuous medium with high electrical conductivity (which includes rocks), in this frequency range, has a relatively low attenuation coefficient, which theoretically should allow for the recording of productive formations at depths of up to 5000 m. With increasing frequency, the amplitude of the natural EM field decreases, which makes it difficult to observe the SE effect at higher frequencies.

The group of authors in [29,30,31] indicated that the amplitude at the station for observing telluric currents and electromagnetic field variations is increased before an earthquake, which apparently can be used to predict earthquakes. In the opinion of the authors of this article, it is interesting to record seismic vibrations and the electromagnetic field before an earthquake, since the influence of this phenomenon on the amplitude of the registered SE effect on the surface is possible. Thus, the observation of the seismoelectric effect at telemetry stations for observing telluric currents and the electromagnetic field may serve as an additional indicator of an earthquake in the future.

In contrast to previously published studies [27,28], this work presents the modeling data and the estimation of the amplitude of the signals of seismic and EM fields in the extended frequency range from 0.1 Hz along the lower boundary. For this purpose, the SE effect observation system was implemented, in contrast to earlier experiments, on molecular fluid seismic receivers with a higher sensitivity including in the range from 0.1 Hz to 100 Hz. In order to experimentally verify the effectiveness of molecular sensors, the article presents new data on measurements of the SE effect on a laboratory bench as well as on a real hydrocarbon field. Based on the results of the experiments carried out at the gas condensate field, a comparative analysis of the effectiveness of the use of molecular geophones with the option of using induction sensors of seismic vibrations was made.

## 2. Materials and Methods

We employed a simplified approach for time-domain numerical simulation of the seismoelectric field, implying the successive solution of three connected partial differential equation (PDE) problems instead of a single fully coupled mechanical-electromagnetic problem similar to the one analyzed in [4]. Those include the equation of motion for the isotropic elastic medium (a), Frenkel equation for the pore pressure assuming poroelastic Biot model (b), and finally, Maxwell’s equations governing electromagnetic field components in quasistationary approximation (c).

(1) $$\rho_{c}\frac{\partial^{2}\vec{u}}{\partial t^{2}}-\nabla \cdot \tau =\vec{F}.$$

Problem (1) is solved in terms of the displacement vector $\vec{u}\;$= $\vec{u}$(*x, y, z, t*) describing the motion of the piecewise-uniform isotropic elastic medium, characterized by three geomechanical constants: bulk modulus *K*, shear modulus *G*, and density *ρ*_c_. For simplicity, we restricted ourselves to considering the two-dimensional (2-D) structure with the modeling domain and the displacement field components limited to the coordinate plane (*x, z*). In (1), τ denotes the stress tensor, and ***F*** stands for external force. We assumed *F* in the form of vertical time-dependent force, applied at the lateral boundary of modeling region to simulate natural microseismic wave propagating through the medium, while its amplitude variation was specified based on the *z*-component of the measured microseismic timeseries *v*(*t*):

*F_z_*(*x* = 0, *z*, *t*) = *v*(*t*). (2)

Once problem (1) is solved, the obtained displacement field {*u_x_, u_z_*} is used to calculate the volume strain

(3) $$\vartheta =\frac{\partial u_{x}}{\partial x}+\frac{\partial u_{z}}{\partial z},$$

which is then used as input to problem (4):

(4) $$\frac{1}{K_{1}}\frac{\partial^{2}p}{\partial t^{2}}+\frac{\beta -1}{\beta^{\prime }}\frac{\partial^{2}\vartheta }{\partial t^{2}}=\frac{1}{\beta^{\prime }\rho }\nabla^{2}p-\frac{\eta }{k_{p}\rho }\left(\frac{1}{K_{1}}\frac{\partial p}{\partial t}+\frac{\beta }{\beta^{\prime }}\frac{\partial \vartheta }{\partial t}\right).$$

Problem (4) is a well-known Frenkel equation governing the propagation of the longitudinal pore pressure waves in fluid-saturated poroelastic rock [1]. It is formulated with respect to the perturbations of the pore pressure *p* = *p*(*x, z, t*), arising in response to mechanical deformation, described here in terms of volume strain *ϑ*(*x, z, t*). Pore pressure equation coefficients are dependent on material properties including the bulk moduli of the dry porous rock (*K*), pore fluid (*K_l_*), rock matrix (*K_s_*), fluid density (*ρ*), and viscosity (*η*) as well as rock permeability *k_p_*, a factor relating the fluid flux and hydraulic gradient according to the Darcy’s law. The quantities β and β՛ are calculated from these parameters and porosity coefficient (n) as

(5) $$\beta =\left(1-\frac{K}{K_{S}}\right)\frac{1}{n},\;\beta^{\prime }=1+(\beta -1)\frac{K_{l}}{K_{S}},$$

Thus, the pore pressure wave field is modeled in time domain through solving (1) for a specific set of boundary conditions either in terms of external force or displacement variation and substituting the resulting volume strain time-derivatives $\frac{\partial \vartheta }{\partial t},\;\frac{\partial^{2}\vartheta }{\partial t^{2}}$ calculated over the entire modeling domain, into (4). This is followed by recalculation of the time-varying pore pressure distributions into the electrokinetically-generated electric field **E**_ek_*,* by making use of the Helmholtz–Smoluchowski equation:

(6) $${\vec{E}}_{\mathrm{ek}}=-c\cdot \nabla p\cdot (x,\;z,\;t),$$

where *c* is the so called streaming potential, most rocks having values around 10^−6^–10^−7^ V/Pa. The latter can be converted into external current density:

(7) $${\vec{j}}_{\mathrm{ext}}=\sigma {\vec{E}}_{\mathrm{ek}},$$

where σ is the electrical conductivity of the medium.

Thus, knowing pore pressure gradient, one can calculate the external electrical current being the source of the seismoelectric field and having non-zero values within fluid-saturated regions of the structure. It is then substituted into the right-hand side of Maxwell’s equations relating electric and magnetic field vectors $\vec{E}\left(x,z,t\right)$ and $\vec{H}\left(x,z,t\right)$:

(8) $$\{\begin{array}{c} \mathrm{rot}\vec{H}=\sigma \vec{E}+{\vec{j}}_{\mathrm{ext}}\\ \mathrm{rot}\vec{E}=-\mu \frac{\partial \vec{H}}{\partial t} \end{array}$$

where μ is the magnetic permeability of free space. The EM problem is formulated assuming in-plane field geometry.

All three PDE problems, starting from linear elasticity Equation (1), poroelasticity fluid flow problem (4), and electromagnetic (EM) problem (8) are solved numerically using the finite element method (FEM), common triangular 2-D mesh, and consistent time-stepping. Thus, the combination of (2), (4), and (8), related by (3) and (6) and (7) yielded the solution in terms of electric field strength variation.

Although the above formulation neglects the effect of pore pressure on elastic strain, which means no (slow) Biot waves arise in the simulated field [5], the scheme is sufficient to understand the basic features of the microseismicity-associated SE signals, allowing fast time-domain computation.

**Figure 1:**
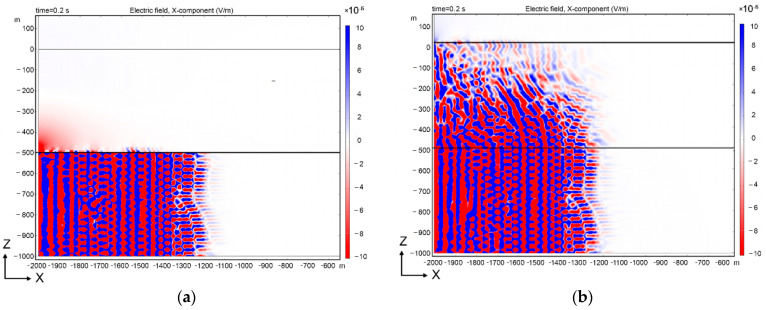
Distribution of the electric field strength in a two-layer medium during the propagation of a microseismic wave with a flat front (from left to right). Only the bottom layer (**a**), or both layers (**b**) are considered fluid saturated.

Figure 1 shows the simulation data of the seismo-electric effects for a poroelastic medium according to the above Equations (1)–(8). In this case, the amplitude of the induced field Ex is shown. The calculation is given for two cases when a layer from 0 to −600 m is not a poroelastic medium (Figure 1a) and when it is (Figure 1b). The implementation of white noise in the frequency band 0.1–100 Hz was used as the emitting signal.

**Figure 2:**
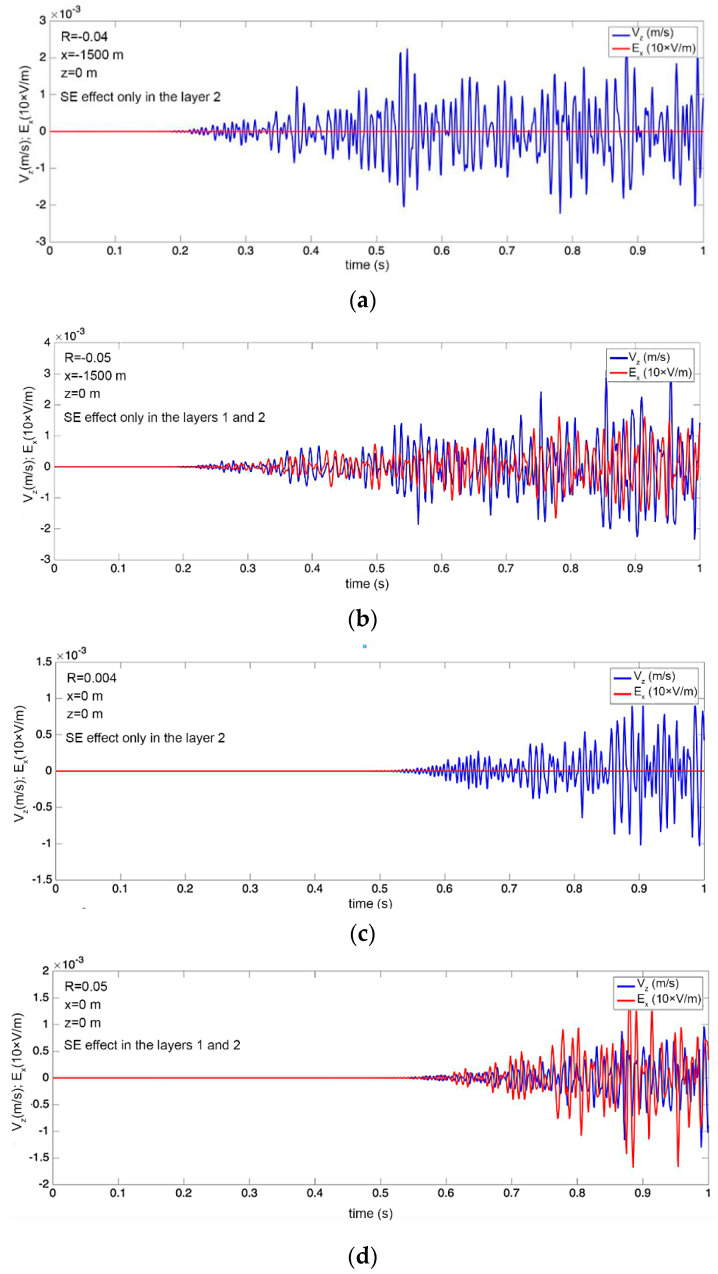
Simulated time series of the vertical velocity and the horizontal component of the electric field at two points on the day surface. (**a**) Figure at the coordinates x = −1500, z = 0 layer where there is the poroelastic medium (up z = −500 to z = −1000) in which there is a SE effect, (**b**) where there is the poroelastic medium(up z = −0 to z = −1000) in which there is a SE effect, (**c**) figure at the coordinates x = 0, z = 0 layer where there is a poroelastic medium (up z = −500 to z = −1000) in which there is a SE effect, (**d**) the poroelastic medium (up z = −0 to z = −1000) in which there is a SE effect.

Figure 2 shows the timing diagrams of the simulated signals on the day surface at different observation points. The simulated wave propagates from left to right (Figure 1). The data obtained show the presence of a correlation between the recorded EM and seismic fields on the daytime surface of the Earth at the coordinates x = 0, z = 0, and x = −1500, z = 0 (for the case when both layers are considered fluid-saturated). If only the lower layer is fluid-saturated, then the amplitude of the EM field on the day surface will be of the order of E = 0.1 μV/m.

As a result of modeling, it was shown that the registration of seismoelectric effects is possible at depths of both 500 m and deeper, since the obtained values of the Ex amplitude for a depth of 500 m were of the order of 10^−3^ V/m. Experimental observations at fields with a depth of 2000 m show that the Ex amplitude was on the order of 1–20 μV/m, which is consistent with the modeling data and theoretical estimates.

**Figure 3:**
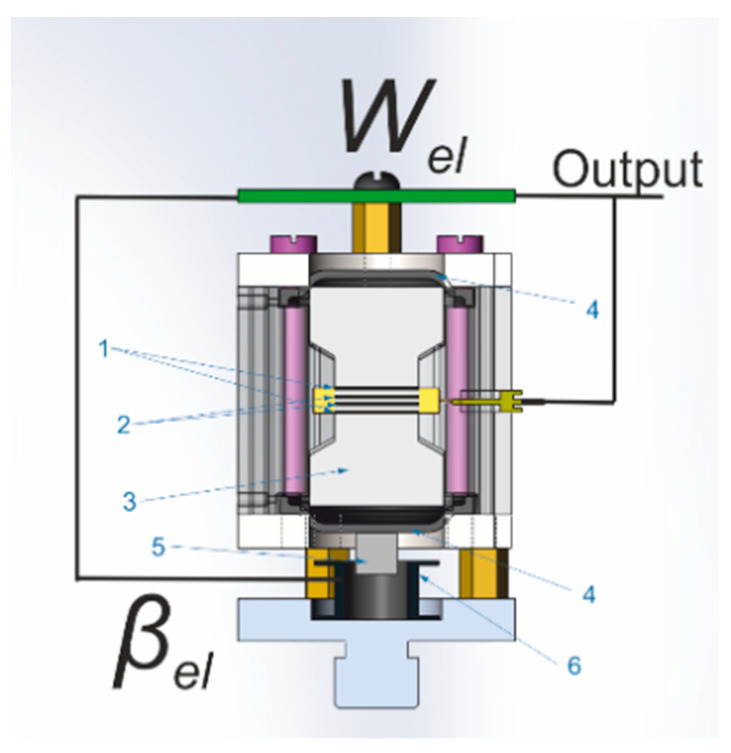
Schematic representation of a molecular electronic (ME) sensor with an electromagnetic power feedback system: 1—anodes; 2—cathodes; 3—electrolyte; 4—membranes; 5—magnet; 6—electromagnetic coil.

We chose a sensor to work as a geophone with increased sensitivity, the schematic design of which is shown in Figure 3. The sensing element is a liquid-permeable four-electrode electrochemical cell located in a housing filled with a highly concentrated electrolyte solution 3. The ends of the housing are limited by flexible membranes 4. Magnet 5 and coil 6, shown of Figure 3, form a mechanism for generating a feedback signal. The principle of operation is that the forces of inertia associated with ground vibrations set the fluid in motion. In turn, the flow of liquid through the conversion element changes the electric current flowing between the electrodes. The current variations are converted into an output voltage with a transfer function W_el_ at the outputs, which controls the feedback mechanism via a separate electronic stage with a transfer function.

In fact, this solution is an analogue of well-known broadband seismometers with electrodynamic feedback [32], with the difference being that instead of a capacitive transducer, its molecular-electronic analogue is used. In terms of sensitivity, we can talk about the equivalence of molecular-electronic and capacitive converters. At the same time, the molecular-electronic converter, in contrast to the capacitive one, does not contain moving elements, which for the normal operation of the device must be positioned with high accuracy relative to each other. This makes the device cheaper and less demanding in terms of transportation and installation, due to which it is widely used in the field [33,34,35,36,37].

The transfer function of the described device with feedback, defined as the ratio of the output voltage U_out_ to the effective acceleration a, can be represented as:

(9) $$W=\frac{U_{\mathrm{out}}}{a}=\frac{W_{\mathrm{sens}}W_{\mathrm{el}}}{1+W_{\mathrm{sens}}W_{\mathrm{el}}\beta_{\mathrm{el}}}$$

where W_sens_ denotes the transfer function of the sensor, which includes both its mechanical system and the electrochemical conversion element.

The feedback electronics in the operating frequency range is a differentiating chain, which, according to (9), under the condition of deep feedback W_sens_ W_el_ β_el_ >> 1 gives U_out_ = a/(iω) and the output signal is proportional to the integral of the acceleration (i.e., oscillation speed).

**Figure 4:**
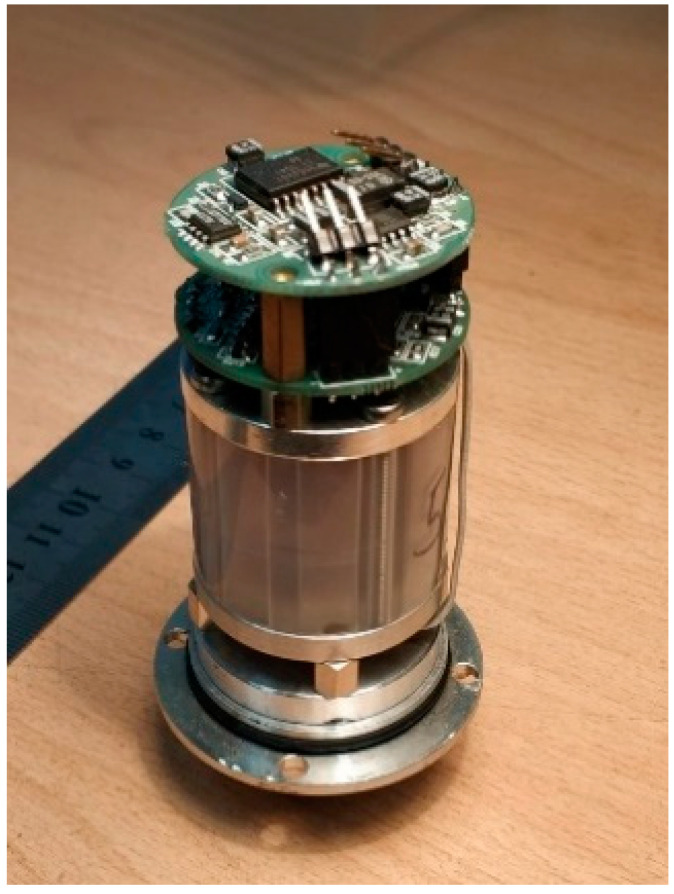
The internal structure of the molecular electronic seismometer MTSS-1001 WB used in this work.

To carry out experimental studies in this work, we used a single-component broadband molecular electronic seismometer MTSS-1001 WB, the appearance of which is shown in Figure 4 without an external case to illustrate its internal structure. The technical characteristics are given in Table 1. For comparison, there are known devices that use liquid and solid-state microstructures as a sensitive element, which practically implement the molecular-electronic principles of signal conversion (MET means molecular-electronic transfer) [38,39,40,41,42].

**Figure 5:**
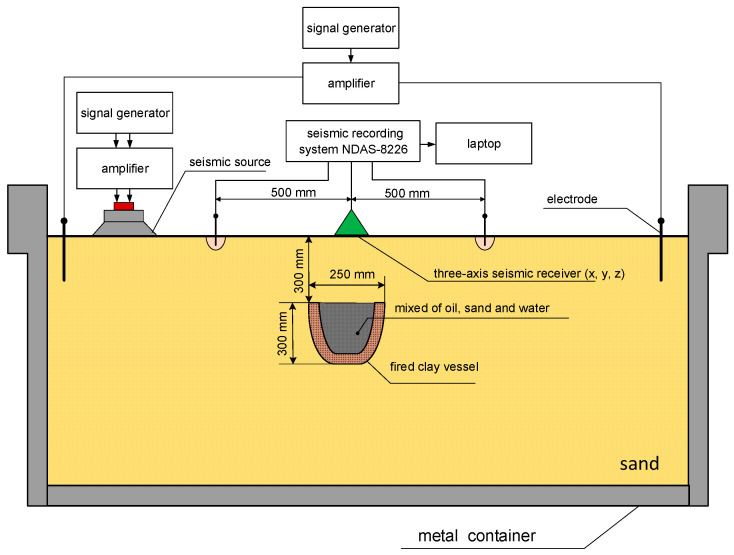
Scheme of tank measurements.

**Figure 6:**
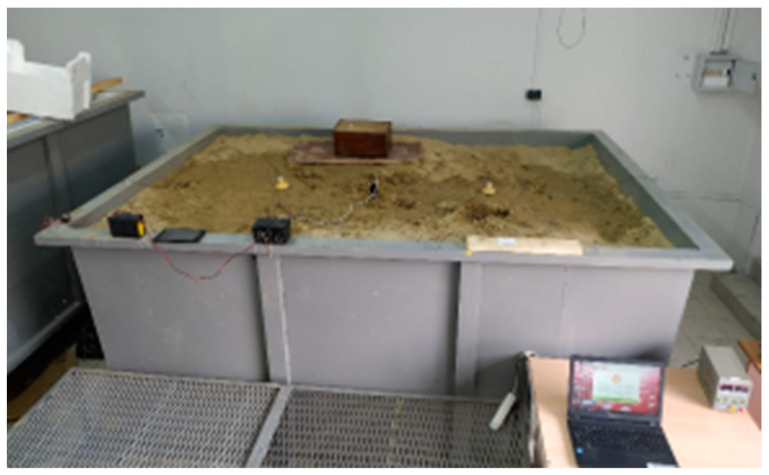
The external view of the measuring stand.

Laboratory studies were carried out to verify the results of the theoretical estimates and simulation results. The measurement scheme is shown in Figure 5. For this purpose, a special measuring stand was made on the basis of a metal basin with a volume of V = 8 m^3^, filled with a sand-clay mixture. The external view of the measuring stand is shown in Figure 6.

Physical modeling of the SE effects was carried out as follows. A reservoir of fired porous clay with a mixture of water, sand, and petroleum products was placed at a depth of h = 30 cm. The simulation of currents flowing through the medium was realized using grounded electrodes connected to a noise-like signal generator. Seismoacoustic signals were excited using acoustic emitters (i.e., low-frequency speakers) (Figure 5). To equalize the amplitude of the emitted signal at low frequencies, a software-implemented spectrum correction was used. The signals emitted by the noise generator were completely uncorrelated, which was experimentally tested on a tank with a sandy-clay mixture without a simulator of a hydrocarbon reservoir. When the noise signals were excited, the maxima of the cross-correlation function were absent.

Subsequently, we conducted field experiments on a real field. The observations were carried out on an area for which there was data on explored gas reserves obtained in the course of geophysical exploration using standard non-explosive common depth point method (CDPM) of 2D seismic exploration as well as data of long-term observations (2014–2020) of the passive SE effect over this gas condensate field, obtained using standard induction geophones with a frequency range from 10 Hz to 100 Hz and a sensitivity of 85 V/(m/s).

**Figure 7:**
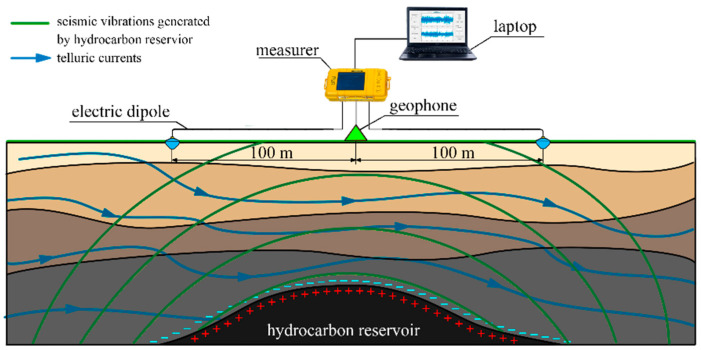
Scheme of measurements by a passive seismoelectric method.

To analyze the effectiveness of the use of molecular seismic receivers to increase the informativity of the passive SE observations by expanding the lower limit of the frequency range to 0.1 Hz as well as increasing the sensitivity of the seismic vibration sensor to 240 V/m/s (see Table 1), two series of experimental field work were carried out in July 2019 and August 2020, respectively. The experiments were carried out in the same field in the used cases of both molecular geophones and induction sensors. At the same time, as induction geophones, the same ones in the experiments on observing the SE phenomena (2014–2018), namely GS-ONE [43], were used. The measurement scheme is shown in Figure 7 [44,45].

It should be noted that in the GS-ONE geophone application, a seismic streamer of N = 24 geophones with a length of 200 m was used. Thus, the signal-to-noise ratio was increased to the value:

(10) $$\mathrm{SNR}=\sqrt{N}\approx 4.89$$

In the case of molecular geophones, only one seismic sensor was used to register the SE effect, connected to the NDAS-8226 recorder.

**Figure 8:**
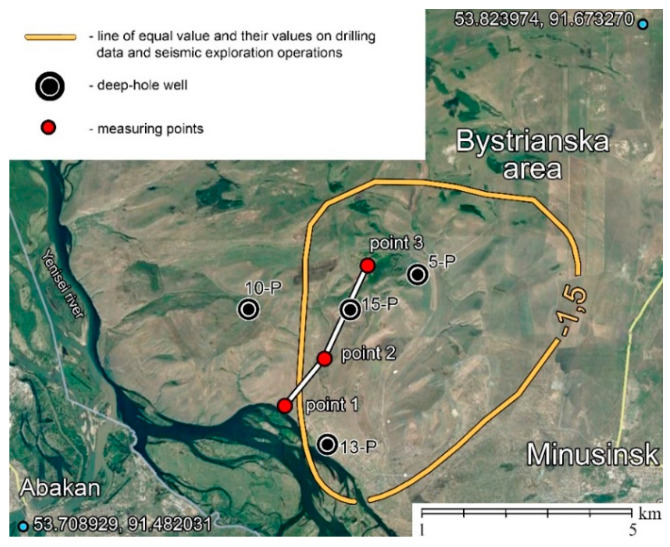
Map of the experimental field work.

**Figure 9:**
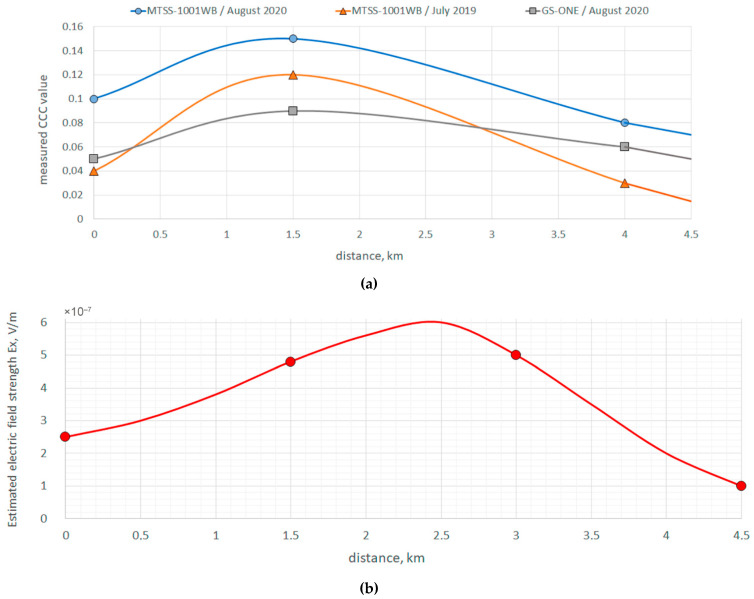
Averaged values of cross-correlation coefficient (CCC) obtained in 2019–2020 including when using new equipment (**a**), and the calculated graph of the electric field strength above the reservoir model (**b**).

The experiments were carried out for the purpose of comparative analysis of the efficiency of using molecular sensors and induction sensors, which we used in the framework of observations in previous years to implement the optimal equipment for the hydrocarbon search method based on the passive SE method. Figure 8 shows a map of the implementation of works, while Figure 9 shows the results obtained in comparison with the measurements in the previous years.

The measurement time at each point in different years was T = 180 s. The receiving electrodes were dipole antennas L = 100 m long with non-polarizable electrodes at the ends, which are ceramic flasks filled with a solution of copper sulfate with a copper electrode immersed in them. This design avoids the parasitic effect of polarization at the interface between the electrode and ion-conducting medium, which will be an additional source of noise.

## 3. Results

In the course of experimental modeling on a laboratory bench (Figure 6), it was found that the registration of such effects on a sand tank using molecular seismic field sensors and a specialized seismic station NDAS-8226 was not possible, since the size of the simulated field was incommensurably small in relation to the recorded effects, and the sensitivity of this equipment and the seismic receivers was not sufficient to register the SE effects on a sand tank. The results obtained on the observation of SE under laboratory conditions agreed with the works of other authors [13,14], in which the effect was recorded under laboratory conditions at higher frequencies of 10–150 kHz. The experiment was carried out in order to check the registration of SE effects at frequencies up to 100 Hz, since in field conditions, this frequency range is the most informative [17,18]. However, at present, the results of laboratory experiments in this range have not been presented. The scientific novelty and importance of the results obtained lies in the fact that they make it clear to researchers that the verification of low-frequency models from 0.1–100 Hz is possible only in the field on real hydrocarbon fields. Additionally, as a result of laboratory experiments, the equipment for the SE observations was calibrated. It was found that there are no parasitic maxima of the CCF coefficient between EM noise and microseisms. This confirmed the correct operation of the measuring equipment and its readiness for carrying out field experiments in a hydrocarbon field.

In [1,4,5,8,13,14], an attempt was made to theoretically and experimentally substantiate the SE effect, described by Frenkel, on the basis of the accumulated modern knowledge in the field of the electrokinetic theory of electrolytes, linking the rheological, elastic parameters of the medium (including the porosity and fluid saturation of rocks) with the magnitude of the induced electric field. As an experimental check of the SE effect, a physical model based on the measurement of the SE effect in the ultrasonic range is proposed. At present, there is no experimental verification of this approach in the field, and in the author’s opinion, its applicability for hydrocarbon prospecting is questionable.

In real fields, an increase in the sensitivity of the receiving equipment will increase the exploration depth and detection of smaller hydrocarbon deposits, therefore, the use of more modern seismic receivers, together with analog band amplifiers, will increase the final sensitivity.

Figure 9 shows the observation profile, which was measured in 2019–2020. According to the data obtained during the exploration drilling, the field is located approximately at a depth of 2000 m (or, according to seismic data, two-way time ≈ 1.5 s). Well 15-P, marked in the figure, has a gas outlet, and is currently mothballed. This field is well studied and is a testing ground for developing new methods of hydrocarbon prospecting as it has easy transport accessibility.

In Figure 9, the values obtained by measuring the seismoelectric coefficient are indicated for point 1—a distance of 0 m, point 2—a distance of 1500 m and point 3—a distance of 4000 m. The values are given for different measuring sensors. Figure 9b shows the calculated data of the electromagnetic field for this field obtained in [25]. According to the obtained curve, the distribution of the amplitude of the electric component Ex of the electromagnetic field can be found from the curve. According to the authors, this curve is consistent with the data of the experimental observations, although the estimated maximum was observed at a distance of 2500 m, while the experimental data showed a maximum at a distance of 1500 m. In this case, additional observations are required between 1500 and 2500 m, which will allow a more accurate comparison of the calculated values of Ex and the observed values of the seismoelectric effect.

To analyze the effectiveness of the application of molecular-electronic sensors of seismic vibrations in comparison with induction geophones GS-ONE, three characteristic points on the gas condensate area were selected (Figure 8):

1. Outside the boundaries of the field (point #1—53.7347459, 91.5747232);
2. On the border of the field (point #2—53.747398, 91.582634);
3. In the center of the field (point #3—53.768098, 91.597226).

Table 2 shows the values of *K* (maximum of the cross-correlation function) for two different systems for observing the SE effect, which were obtained from the results of the experiments carried out in 2019 and 2020.

As shown in Table 2, obtained in 2019–2020, the experimental data confirmed the results of the measurements in 2014–2018 [27,28,44,45,46,47]. Figure 9b also shows a curve obtained by solving Equations (1)–(8), according to which the values of the electric field strength E will correspond to the magnitude of the observed SE effect on the day surface. Within the framework of a model experiment, we calculated the values of Ex at points 0, 1500, 3000, and 4500 m. From Figure 9b, the maximum manifestation of the SE effect is at a distance of 2500 m. The experimental shape of the curve (Figure 9a) at points 0, 1500, and 4000 m corresponded to the simulation results at these points. The discrepancy between the maximum of the experimental (1500 m) and theoretical (2500 m) curves can be explained by the fact that at point 2500 m, there was no possibility of taking measurements due to the difficult terrain (rocky ravines with steep slopes).

Earlier [27,28,46,47], we have shown, both theoretically and empirically, that the SE effect of the second kind is most pronounced at the edges of the field. This is due to the distribution function of the electric charges along the boundaries of the field, which cause an EM field when exposed to microseismic disturbances. As shown in Table 2, the experimental data obtained in 2019–2020 confirm the measurement results of 2014–2018 as well as those developed on the basis of the solution of (1)–(8), the theoretical models of SEE (simulation results are graphically presented in Figure 9b).

## 4. Discussion

Analyzing the results obtained using a seismic streamer consisting of 24 GS-ONE geophones and recording the SE signals obtained on the basis of the NDAS-8226 registration system with one molecular electronic sensor MTSS-1001 WB with the characteristics presented in Table 1, the following conclusions can be drawn. Despite the fact that in the first case, a group of geophones was used, thereby increasing the SNR by 4.89 times, the value of *K* at each of the three observation points (in 2020) in the case of using the molecular-electronic sensor MTSS-1001 WB turned out to be higher by 30–100%. The ratio of the sensitivity parameters of the GS-ONE and MTSS-1001 WB geophones is:

(11) $$\frac{S_{NDAS}}{S_{GS}}=\frac{250\;V/(m/s)}{85\;V/(m/s)}=2.94$$

and the use of a group of 24 GS-ONE geophones allows raising the SNR to *N* = 4.89. The SNR value is given for the ideal case, when the length of the field is L >> l (length of the seismic streamer) and the anisotropy of the geological environment is not taken into account. Despite this, according to Table 2, the system based on molecular electronic geophones provided a more informative picture in terms of SEE observation. Therefore, it would be logical to assume that the increase in information content in this case is provided not only by increasing the sensitivity of the sensors, but also by expanding the analyzed frequency range in the lower frequency range, up to 0.1 Hz from 10 Hz, typical for the GS-ONE geophone.

In the course of generalizing the data obtained in different years by the passive SE method shown in the figure, it was revealed that the maximum of the cross-correlation function marks the edges of the field. The line in Figure 9a repeats the shape of the curve obtained when calculating the amplitude values of the horizontal part of the E component [23]. This was the first attempt to process data obtained in different years of observation. Based on the experience of [27,28,44,45,46,47] on this profile, it can be concluded that the amplitude value of the observed cross-correlation coefficient on the Earth’s surface depends on many factors. Thus, the absolute value of this coefficient cannot be used for the assessment, and, therefore, the shape of the resulting curve is a more important factor. Thus, it is necessary to include additional measurements in the work methodology by the passive SE method before starting field work. These measurements are carried out at a predetermined point, where, according to the explored data, there are no productive anomalies, which will make it possible to compare the estimated parameter above the assumed field and at the point where it does not exist, which was done first at the calibration stage in laboratory conditions, and then in the field at aa distance of more than 5 km from the investigated field.

## 5. Conclusions

Analysis of the data obtained in different years showed that there was a repeatability of the measurement results as well as convergence with the results of the mathematical modeling. This indicates the adequacy of the previously proposed mathematical model of a hydrocarbon reservoir for the passive SE method.

The passive version of the SE method allows for registration of the boundaries of the field (i.e., carry out only its contouring without analyzing the geological structure of the layers). For this purpose, there are active methods of seismic sounding of the geological environment. However, even in such a limited application, this method can save money in the exploration of minerals. The use of passive methods is advantageous for work in hard-to-reach areas such as swampy or mountain-taiga areas, where the use of large-sized equipment is impossible.

Thus, the collected statistics on observation points shows the possibility of using this method when carrying out industrial field work in conjunction with standard seismic exploration, since the implementation of the passive SE method requires about three grounded electric dipoles.

The main conclusion that can be drawn from the results of the experimental studies on a hydrocarbon field is that molecular sensors with autocorrelation function in the lower limit of the range from 0.1 Hz, which have a sensitivity higher than that of induction geophones, can significantly expand the information content of the SE method. In addition, as shown in the article, a single MTSS-1001 transducer performed better than a streamer of 24 GS-ONE geophones. This allows us to count on the fact that the use of molecular-electronic geophones will make it possible in the future to create more compact and rapidly deployable equipment for the search for hydrocarbons by the SEE method. A further direction in the development of this method is seen by the authors in the development of optimal algorithms for extracting information from the natural electromagnetic and seismic noises of the Earth.

## Figures and Tables

**Table 1:** Technical characteristics of the seismometer MTSS-1001 WB.

Technical Characteristic	Value
Frequency range	0.1–100 Hz
sensitivity	250 V/m/s
maximum measurable signal	30 mm/s
internal noise	100 nm/s
dynamic range	110 dB
harmonic distortion factor	<1%

**Table 2:** The value of the cross-correlation coefficient *K* according to the SEE observations at the field.

Sensor Type/Year	Point 1	Point 2	Point 3
MTSS-1001WB/August 2020	0.1	0.15	0.08
MTSS-1001WB/July 2019	0.04	0.12	0.03
GS-ONE/August 2020	0.05	0.09	0.06

## Data Availability

The data presented in this study are contained within the article.
